# Ebstein Anomaly: Imaging, Pathophysiology, and Therapeutics

**DOI:** 10.7759/cureus.112612

**Published:** 2026-07-13

**Authors:** Guillermo Cueto-Robledo, Maria-Fernanda Yañez-Marquez, Melissa Garcia-Lezama, Victor-Hugo Ramos-Pacheco, Leslie-Marisol Gonzalez-Hermosillo, Abril-Carolina Mendoza-Lopez, Ernesto Roldan-Valadez

**Affiliations:** 1 Department of Cardiorespiratory Emergencies, Hospital General de México "Dr. Eduardo Liceaga", Mexico City, MEX; 2 Faculty of Medicine, Universidad Nacional Autónoma de México, Mexico City, MEX; 3 Department of Surgery, Centro Médico Nacional La Raza, Mexico City, MEX; 4 Department of Magnetic Resonance, Hospital General de México "Dr. Eduardo Liceaga", Mexico City, MEX; 5 Laboratory of Immunometabolism, Research Division, Hospital General de México "Dr. Eduardo Liceaga", Mexico City, MEX; 6 Faculty of Medicine, Benemérita Universidad Autónoma de Puebla, Puebla, MEX; 7 Department of Research, Instituto Nacional de Rehabilitación Luis Guillermo Ibarra Ibarra, Mexico City, MEX; 8 Department of Radiology, I.M. Sechenov First Moscow State Medical University, Moscow, RUS

**Keywords:** cardiac catheterization, congenital heart defects, ebstein anomaly, echocardiography, heart defects, mri magnetic resonance imaging, tricuspid valve insufficiency

## Abstract

Ebstein anomaly is a rare congenital malformation defined by failed delamination and apical displacement of the tricuspid valve, which produces an atrialized segment of the right ventricle and variable right ventricular dysfunction. Its clinical expression is wide, ranging from severe neonatal cyanosis and heart failure, which carry high perinatal mortality, to incidental detection in adulthood. Because anatomy and physiology vary widely among patients, imaging is central to diagnosis, risk stratification, and treatment planning. Transthoracic echocardiography remains the first-line study and establishes the diagnosis through apical leaflet displacement, leaflet tethering, and right atrial dilation; three-dimensional and transesophageal echocardiography refine the assessment of leaflet morphology and the atrialized right ventricle. Cardiac magnetic resonance imaging quantifies right ventricular and right atrial volumes, ejection fraction, regurgitant volume, and myocardial fibrosis, and increasingly informs the timing of surgery; computed tomography is a complement when magnetic resonance is contraindicated or extracardiac anatomy must be defined. Management is guided by current European and North American congenital heart disease guidelines and is matched to symptom burden and anatomy. Transplacental non-steroidal anti-inflammatory drugs may relieve the circular shunt in selected fetuses. Cone reconstruction has become the preferred surgical repair, with valve replacement reserved for valves deemed unsuitable. Catheter-based options, including device closure of interatrial communications, accessory-pathway ablation, and transcatheter tricuspid valve-in-valve implantation, address specific problems in higher-risk patients, although dedicated transcatheter replacement remains investigational in this population. Long-term care depends on serial imaging to detect residual regurgitation, progressive ventricular dysfunction, and arrhythmia. This review summarizes the anatomy, pathophysiology, multimodality imaging, and contemporary therapy of Ebstein anomaly, with emphasis on how imaging findings translate into clinical decisions.

## Introduction and background

Ebstein anomaly is a rare congenital malformation of the tricuspid valve and right ventricle (RV), first described by Wilhelm Ebstein in 1866 [1]. It affects roughly 1 in 20,000 live births and accounts for fewer than 1% of congenital heart defects, yet it carries a clinical weight out of proportion to its frequency: when the diagnosis is made in utero, perinatal mortality has been reported as high as 45% [2,3].

The lesion results from faulty delamination of the septal and posterior (inferior) leaflets of the tricuspid valve from the RV inlet myocardium. The leaflets remain adherent to the ventricular wall and are displaced apically, so that part of the inlet RV is incorporated into the right atrium as a thin-walled, dilated, and dyskinetic "atrialized" right ventricle (aRV). Leaflet tethering, an enlarged and often sail-like anterior leaflet, and the resulting tricuspid regurgitation (TR) are the anatomical core of the disease [4-6].

Clinical expression mirrors this anatomical variability. Severely affected neonates present with cyanosis and heart failure and have a guarded prognosis, whereas mildly affected adults may remain asymptomatic for decades or first present with an arrhythmia [5,7,8]. Across this spectrum, imaging carries the diagnosis, defines severity, and guides the timing and type of intervention [9,10].

This review summarizes the anatomy, pathophysiology, multimodality imaging, and contemporary therapy of Ebstein anomaly. Throughout, the emphasis is practical: how a given imaging finding maps onto a clinical decision for the pediatrician, radiologist, cardiologist, or surgeon who encounters this uncommon but consequential lesion. As a narrative review, the discussion draws on a focused search of PubMed and MEDLINE for English-language articles combining the terms "Ebstein anomaly" with "imaging", "echocardiography", "magnetic resonance", "computed tomography", "surgery", and "transcatheter", supplemented by the reference lists of key articles and current society guidelines; foundational descriptions were retained alongside literature published through 2026.

## Review

Anatomical features

Ebstein anomaly is best understood as a constellation of abnormalities whose combined severity determines presentation [7]. The defining lesion is incomplete delamination of the tricuspid leaflets, which limits leaflet mobility and produces TR [9,11]. The anterior leaflet is typically enlarged and dysmorphic. Apical displacement of the functional tricuspid annulus is the most sensitive and specific sign of the disease; on a four-chamber view, displacement of the septal leaflet relative to the anterior mitral leaflet exceeding 8 mm/m^2^ is diagnostic [5,10].

The atrialized segment that lies between the true annulus and the displaced valve is thin, dilated, and dyskinetic, while the functional RV is reduced in volume and preload [12,13]. Volume overload from TR dilates the right atrium and the aRV, which in turn worsens RV mechanics. A patent foramen ovale or atrial septal defect is present in most patients and permits right-to-left shunting and cyanosis [5,14].

**Figure 1:**
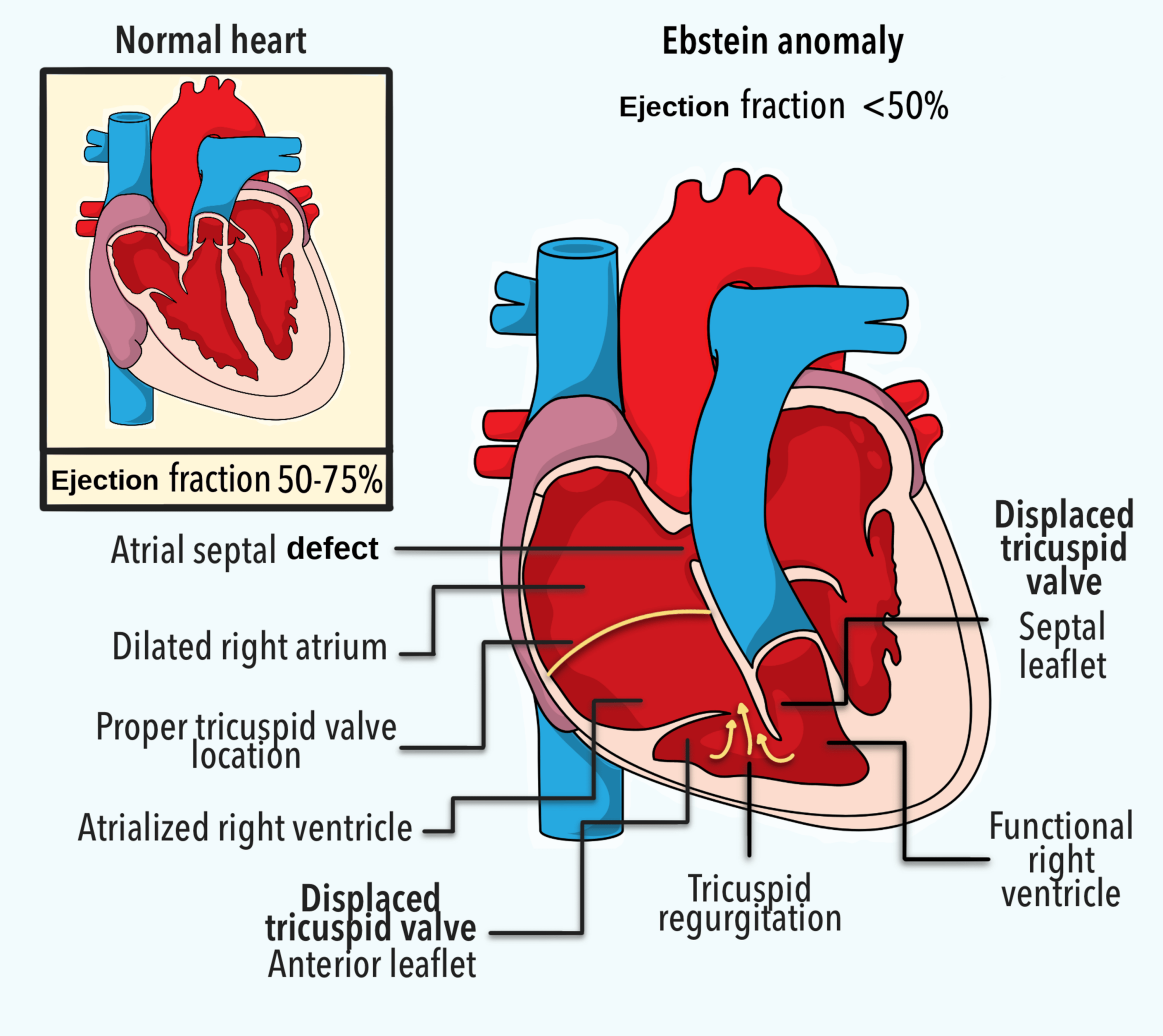
Anatomy of Ebstein anomaly. The schematic compares the normal heart with Ebstein anomaly, labeling the displaced and tethered tricuspid leaflets, the atrialized right ventricle, the dilated right atrium, the atrial septal defect, and the reduced functional right ventricle, with the reduction in ejection fraction indicated. Image created using the Mind the Graph platform (www.mindthegraph.com; CACTUS Communications, Mumbai, India). The diagrams were completely conceptualized and designed by the authors.

**Figure 2:**
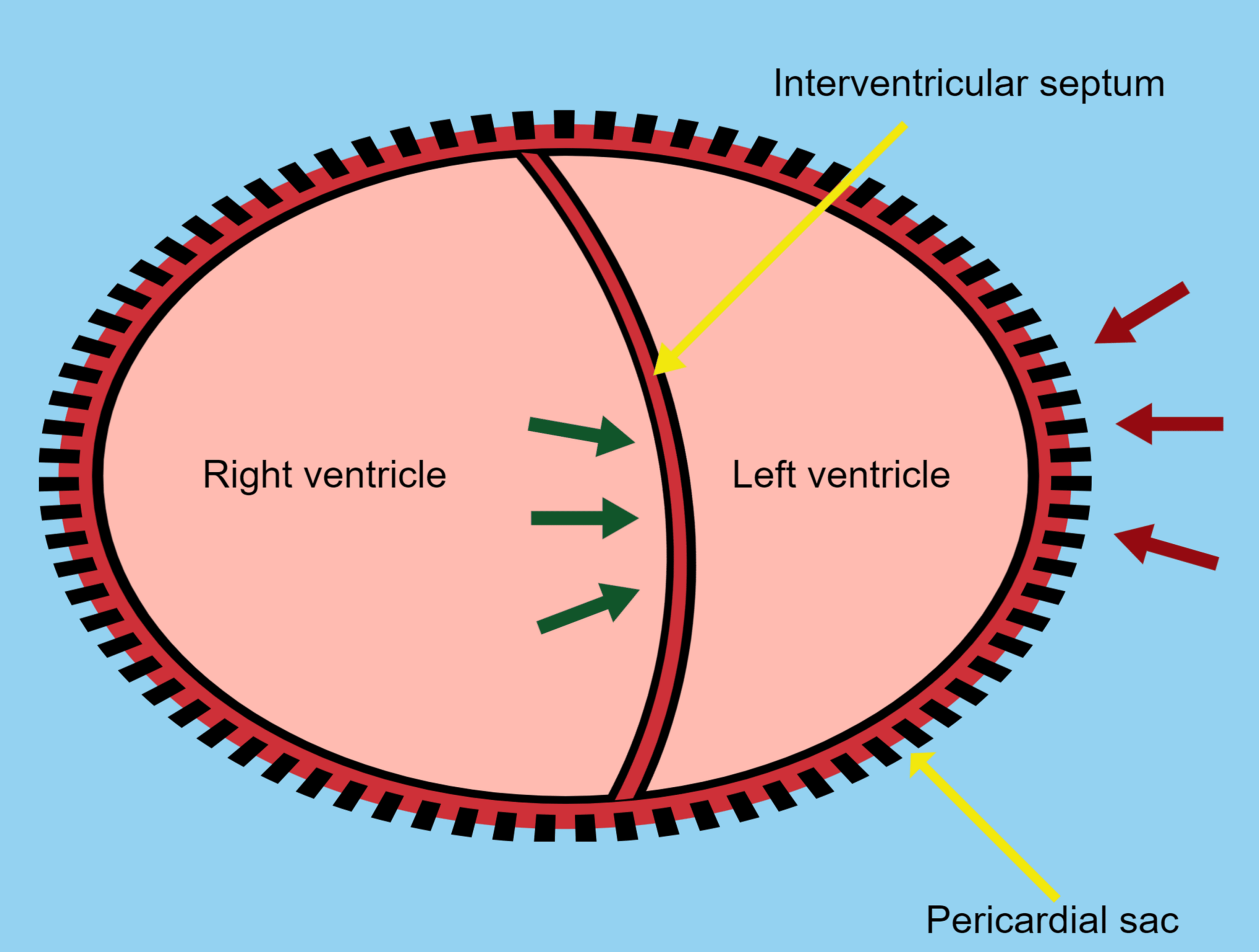
Ventricular interdependence. The dilated right heart and diastolic displacement of the interventricular septum (arrows) compress and constrain the left ventricle, contributing to impaired left ventricular filling and exercise intolerance. Image created using the Mind the Graph platform (www.mindthegraph.com; CACTUS Communications, Mumbai, India). The diagrams were completely conceptualized and designed by the authors.

Although the disease is dominated by right heart pathology, left ventricular (LV) involvement is increasingly recognized in advanced cases. Diastolic displacement of the interventricular septum constrains LV filling and reduces LV systolic performance, a mechanism implicated in the exercise intolerance many patients report [15,16]. Figure 1 depicts the principal anatomical abnormalities, and Figure 2 illustrates how the dilated right heart and a displaced septum compress and constrain the left ventricle.

Ebstein anomaly is frequently accompanied by other lesions that influence management. An interatrial communication, either a patent foramen ovale or a secundum atrial septal defect, is almost universal and underlies cyanosis and the risk of paradoxical embolism; right ventricular outflow tract obstruction or functional pulmonary atresia may coexist, and left-sided abnormalities, such as mitral valve anomalies or LV non-compaction, are also described [14,17]. Accessory conduction pathways, discussed below, are part of the same developmental disturbance. Recognizing these associations at diagnosis matters because each may require dedicated imaging and may alter the surgical or interventional plan [18].

Pathophysiology

The hemodynamic problem in Ebstein anomaly begins with TR and the resulting volume overload of the right atrium and aRV, which lowers effective forward output [5]. The direction of flow across the pulmonary valve, whether antegrade or retrograde, is an important prognostic marker, particularly in the fetus and neonate, where functional pulmonary atresia and a circular shunt can develop [19,20].

Goleski and colleagues quantified the left-sided consequences, reporting lower LV ejection fraction in patients than controls (41% versus 57%) [16]. More recent work indicates that left, rather than right, ventricular status best discriminates patients who develop heart failure, and that LV global longitudinal strain outperforms ejection fraction for prognostication [15,21].

**Figure 3:**
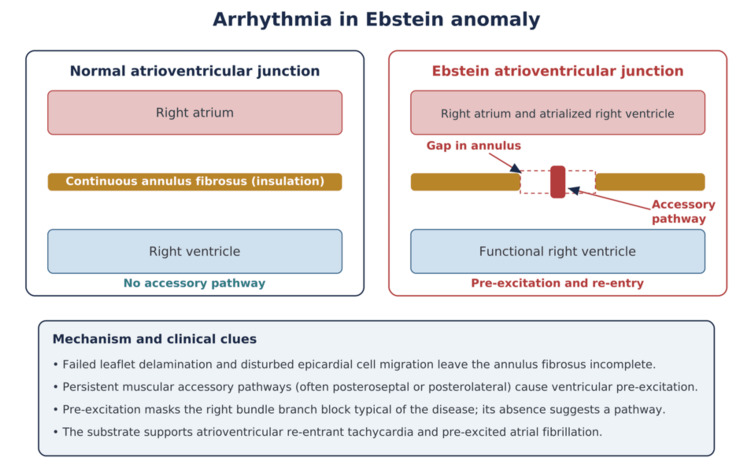
Mechanism of arrhythmia. Incomplete formation of the annulus fibrosus, related to abnormal leaflet delamination and disturbed epicardial cell migration, leaves a gap across the atrioventricular junction through which a muscular accessory pathway persists, typically in a posteroseptal or posterolateral position. The resulting ventricular pre-excitation supports re-entrant tachycardia; because pre-excitation masks the right bundle branch block otherwise typical of the disease, its absence on the electrocardiogram suggests an accessory pathway. Image created using the Mind the Graph platform (www.mindthegraph.com; CACTUS Communications, Mumbai, India). The diagrams were completely conceptualized and designed by the authors.

Arrhythmia is intrinsic to the disease and directly results from abnormal development of the atrioventricular junction. Normally, the annulus fibrosus electrically insulates atria from ventricles; it forms through the migration of epicardium-derived cells. In Ebstein anomaly, abnormal leaflet delamination and disturbed epicardial cell migration leave gaps in this fibrous ring, allowing muscular accessory pathways to persist across the atrioventricular junction, producing ventricular pre-excitation of the Wolff-Parkinson-White (WPW) type [4]. Because myocardial fibers bridge the junction in the malformed, atrialized region, accessory pathways are most often right-sided, in the posteroseptal or posterolateral positions, and multiple pathways are common [22,23]. A useful electrocardiographic clue is that pre-excitation masks the right bundle branch block otherwise typical of the disease; the absence of right bundle branch block therefore raises suspicion of an accessory pathway. These substrates support orthodromic atrioventricular re-entrant tachycardia and pre-excited atrial fibrillation, while the aRV adds to arrhythmogenesis through slowed conduction and abnormal repolarization (Figure 3) [4,22].

Clinical presentation and burden of disease

Symptoms can appear at any age and track with the degree of leaflet displacement and right heart dysfunction [18].

Neonates

Neonatal disease is usually severe. Marked leaflet displacement renders the RV ineffective, TR is profound, and systemic venous return is diverted across the atrial septum, reducing pulmonary blood flow [24]. Cyanosis, heart failure, cardiomegaly, and pulmonary hypoplasia are typical, and the most severe fetuses progress to hydrops, a marker of poor outcome [25]. Contemporary registries underscore the burden: in a national analysis of neonates with Ebstein anomaly, in-hospital mortality remained substantial despite modern care, and fetal series report perinatal mortality approaching one half in the most affected pregnancies [2,26].

Adults

**Figure 4:**
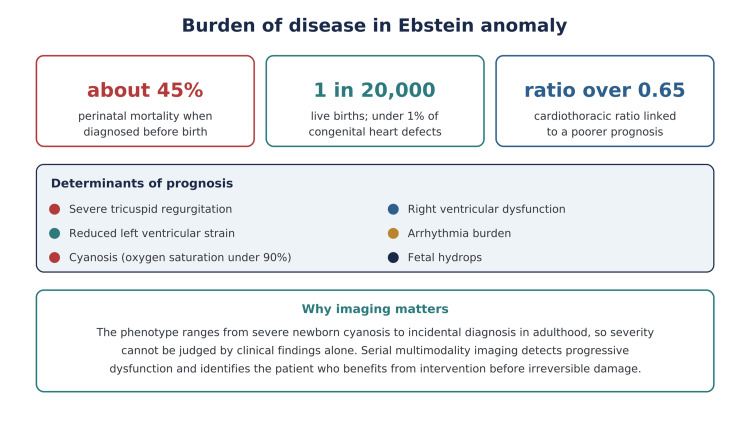
Burden of disease and rationale for imaging. Perinatal mortality reaches approximately 45% when the diagnosis is made in utero, and prognosis is determined by the severity of tricuspid regurgitation, right and left ventricular dysfunction, arrhythmia, and cyanosis. Because severity cannot be judged clinically alone, serial multimodality imaging is used to detect progressive dysfunction and to identify patients who benefit from intervention. Image created using the Mind the Graph platform (www.mindthegraph.com; CACTUS Communications, Mumbai, India). The diagrams were completely conceptualized and designed by the authors.

Adults present more indolently, with exertional dyspnea, reduced exercise tolerance, cyanosis, or palpitations, and an arrhythmia is frequently the first manifestation [8,27]. Natural-history data, available since the earliest clinical series, show that survival depends on the severity of TR and RV dysfunction, as well as the arrhythmic burden [28]. This wide spectrum and the fact that complications such as paradoxical embolism and brain abscess can present as the initial event are the central arguments for early and accurate imaging: timely detection of progressive dysfunction identifies patients who will benefit from intervention before irreversible damage occurs (Figure 4) [29,30].

Imaging evaluation

**Figure 5:**
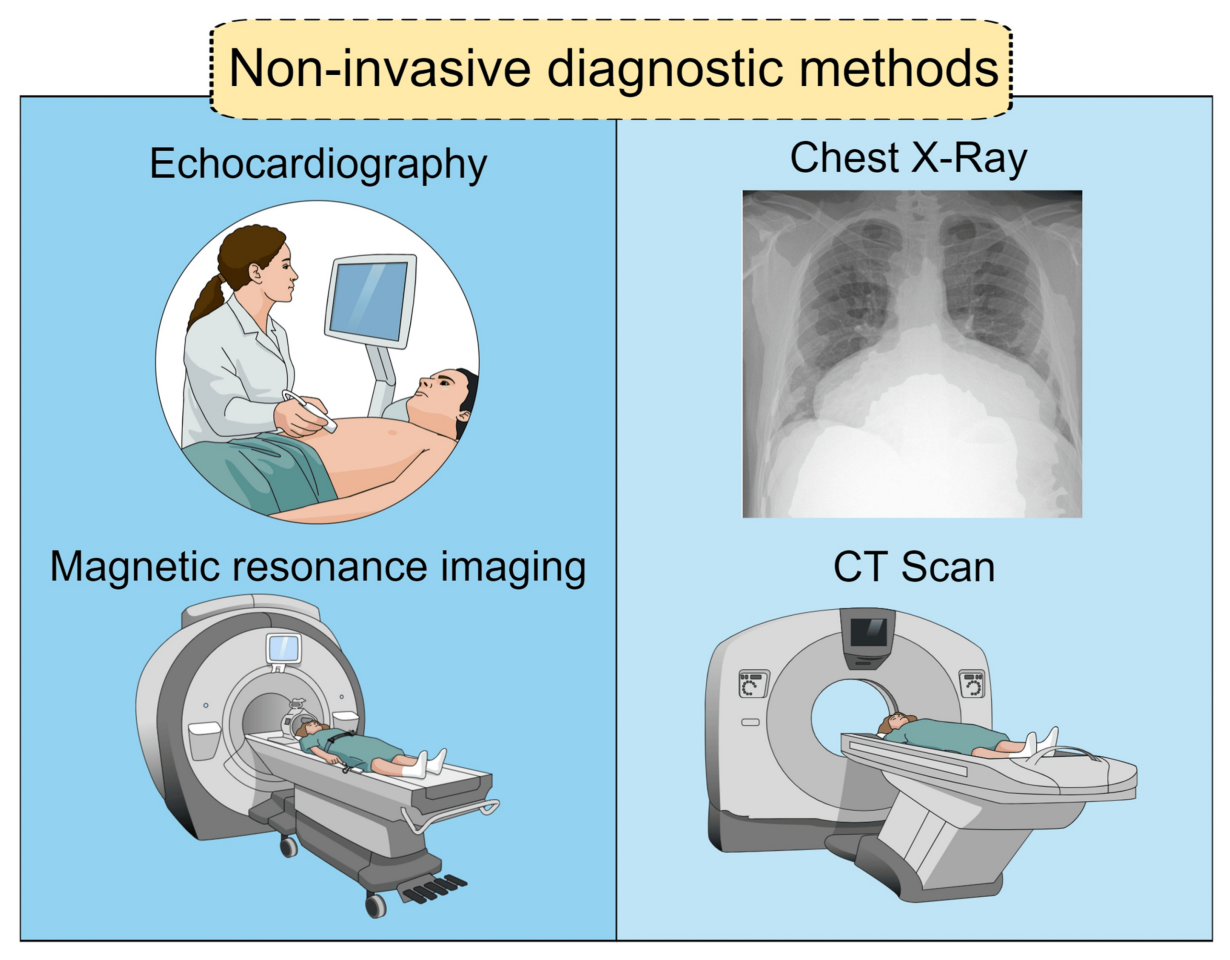
Noninvasive imaging methods. Overview of the noninvasive imaging methods used in Ebstein anomaly: echocardiography, cardiac magnetic resonance imaging, and computed tomography, with their principal contributions to diagnosis and follow-up. Image created using the Mind the Graph platform (www.mindthegraph.com; CACTUS Communications, Mumbai, India). The diagrams were completely conceptualized and designed by the authors.

The diagnosis and serial assessment of Ebstein anomaly rest on a complementary set of imaging tools, each with defined strengths and limitations (Figure 5) [9].

Chest radiography

**Figure 6:**
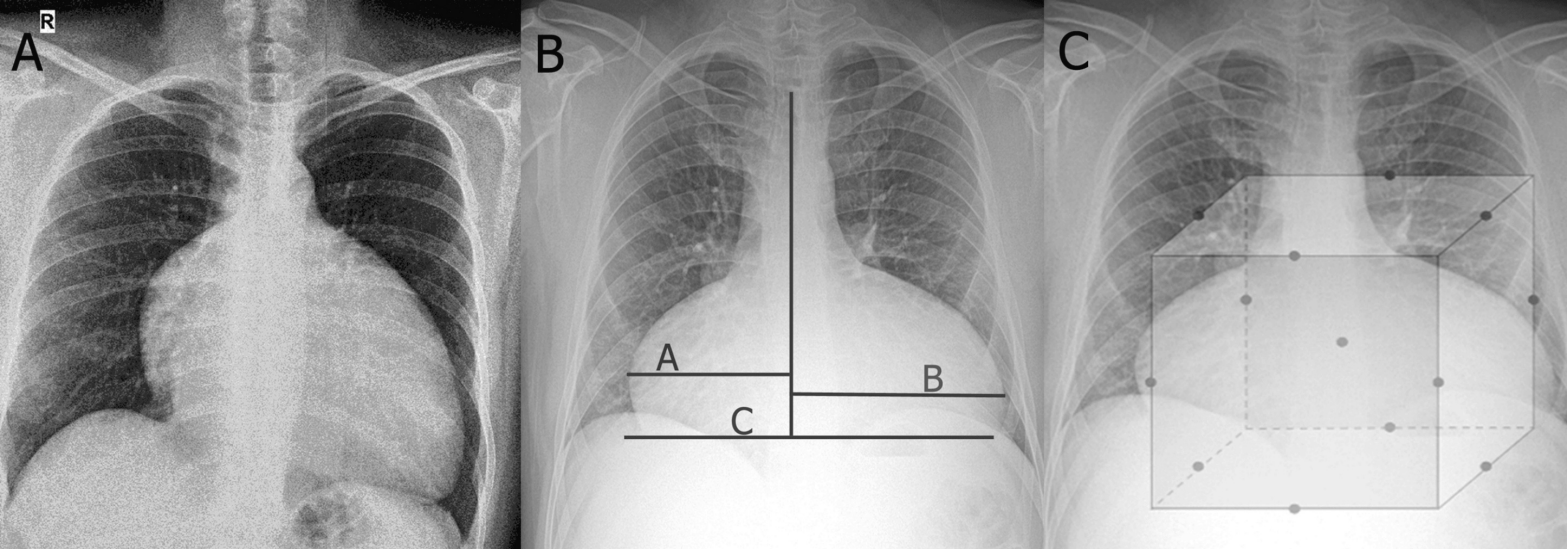
Chest radiographic findings. (A) Globe-shaped cardiomegaly with indistinct great-vessel borders. (B) Increased cardiothoracic ratio. (C) Box-shaped cardiac silhouette with right atrial enlargement, an appearance associated with a poorer prognosis when the cardiothoracic ratio exceeds 0.65.

Although it is not a diagnostic study, the chest radiograph is often the first abnormal investigation. Cardiomegaly with a globular or box-shaped silhouette is characteristic, and a cardiothoracic ratio above 0.65 is associated with a worse prognosis [31,32]. Representative radiographic appearances, including the box-shaped heart and progressive cardiomegaly, are shown in Figure 6.

Echocardiography

**Figure 7:**
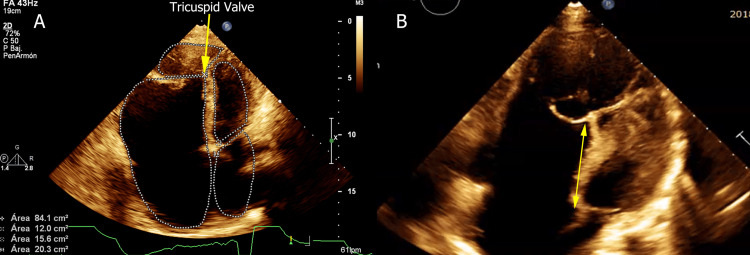
Transthoracic echocardiography. (A) Apical four-chamber view showing low (apically displaced) tricuspid implantation, right atrial dilation, and a small functional right ventricle, with intact interatrial and interventricular septa. (B) Absent delamination of the anterior tricuspid leaflet with right atrial dilation.

Transthoracic echocardiography (TTE) is the reference first-line study and usually establishes the diagnosis. It demonstrates apical displacement and tethering of the tricuspid leaflets, right atrial dilation, and the size of the functional RV. At the same time, color Doppler grades the severity of TR, and agitated saline contrast discloses interatrial shunting [8,17]. The diagnostic displacement is measured on the four-chamber view, relative to the anterior mitral leaflet. These features are illustrated in Figure 7, in which low tricuspid implantation, right atrial dilation, and absent leaflet delamination are evident.

Three-dimensional echocardiography adds en-face visualization of the leaflets and more accurate measurement of the aRV and functional RV areas; the Great Ormond Street (GOS) ratio - defined on a four-chamber view as the combined area of the right atrium and aRV divided by the combined area of the functional RV, left atrium, and left ventricle - is a validated neonatal prognostic index, in which higher grades (GOS grade 3-4) predict markedly worse perinatal outcome [33,34]. Transesophageal echocardiography (TEE) is reserved for inadequate transthoracic windows and for intraoperative guidance, where it resolves posterior leaflet anatomy that determines reparability (Figure 7) [35]. Quantitative echocardiographic parameters, including tricuspid annular plane systolic excursion and fractional area change, are summarized in Table 1, and the Carpentier anatomical classification is given in Table 2.

**Table 1:** Quantitative echocardiographic and cardiac magnetic resonance parameters for assessing right ventricular function and disease severity in Ebstein anomaly. The table lists the principal quantitative indices obtained by transthoracic echocardiography and cardiac magnetic resonance (CMR) imaging, with the aspect of disease that each measure characterizes. The echocardiographic indices capture longitudinal and global systolic function of the functional right ventricle and the severity of tricuspid regurgitation, whereas CMR adds volumetric quantification of the functional and atrialized right ventricle, biventricular ejection fraction, regurgitant volume, and tissue characterization of myocardial fibrosis. Because the geometrically abnormal right ventricle limits the accuracy of any single index, these parameters are interpreted together and tracked serially to inform the timing of intervention. LV: left ventricle; RV: right ventricle; TR: tricuspid regurgitation

Modality	Parameter	Clinical use
Echocardiography	Tricuspid annular plane systolic excursion (TAPSE)	Longitudinal RV systolic function
Echocardiography	Fractional area change of the functional RV (fRV-FAC)	Global functional RV systolic function
Echocardiography	Systolic myocardial velocity of the RV free wall	Tissue-Doppler index of RV function
Echocardiography	Tricuspid regurgitation grade (color Doppler)	Severity of valvular regurgitation
CMR	End-diastolic and end-systolic volumes (EDV, ESV)	Functional RV and atrialized RV volumetry
CMR	Functional RV and LV ejection fraction (EF)	Biventricular systolic function
CMR	Regurgitant volume and late gadolinium/tissue mapping	Quantified TR and myocardial fibrosis

**Table 2:** Carpentier classification of Ebstein anomaly by tricuspid leaflet morphology and right ventricular anatomy. The Carpentier classification stratifies Ebstein anomaly into four anatomical types (A-D) of increasing severity, based on the mobility and displacement of the tricuspid valve leaflets and on the size and contractile function of the atrialized and functional right ventricle (RV). Severity progresses from type A, with a large mobile anterior leaflet and a small contractile atrialized segment, to type D, with extensive leaflet adhesion and near-complete atrialization of a thin, barely contractile ventricle. The classification informs surgical planning, since leaflet mobility and the size of the functional right ventricle determine the feasibility of valve-preserving repair such as cone reconstruction.

Type	Description
Type A	Large, freely mobile anterior leaflet; moderate displacement of septal and posterior leaflets; small, contractile atrialized RV with near-normal functional RV volume.
Type B	Anterior leaflet as in type A; marked displacement of hypoplastic septal and posterior leaflets; larger, non-contractile atrialized RV.
Type C	Restricted anterolateral leaflet motion; significant displacement of posterior and septal leaflets; large non-contractile atrialized RV with a small functional RV.
Type D	Adhesion of anterior, posterior, and septal leaflets; thin ventricular wall with minimal contraction (near-complete atrialization).

Echocardiography can also make the diagnosis before birth. Fetal echocardiography identifies the displaced, regurgitant valve and the dilated right heart, and the presence of a circular shunt, retrograde flow in the arterial duct, and functional pulmonary atresia identifies the fetus at highest risk [20,36,37]. Serial fetal assessment tracks progression and informs counseling and delivery planning, and the degree of cardiomegaly and the presence of hydrops are among the strongest predictors of perinatal death [2,35].

Cardiac magnetic resonance (CMR) imaging

**Figure 8:**
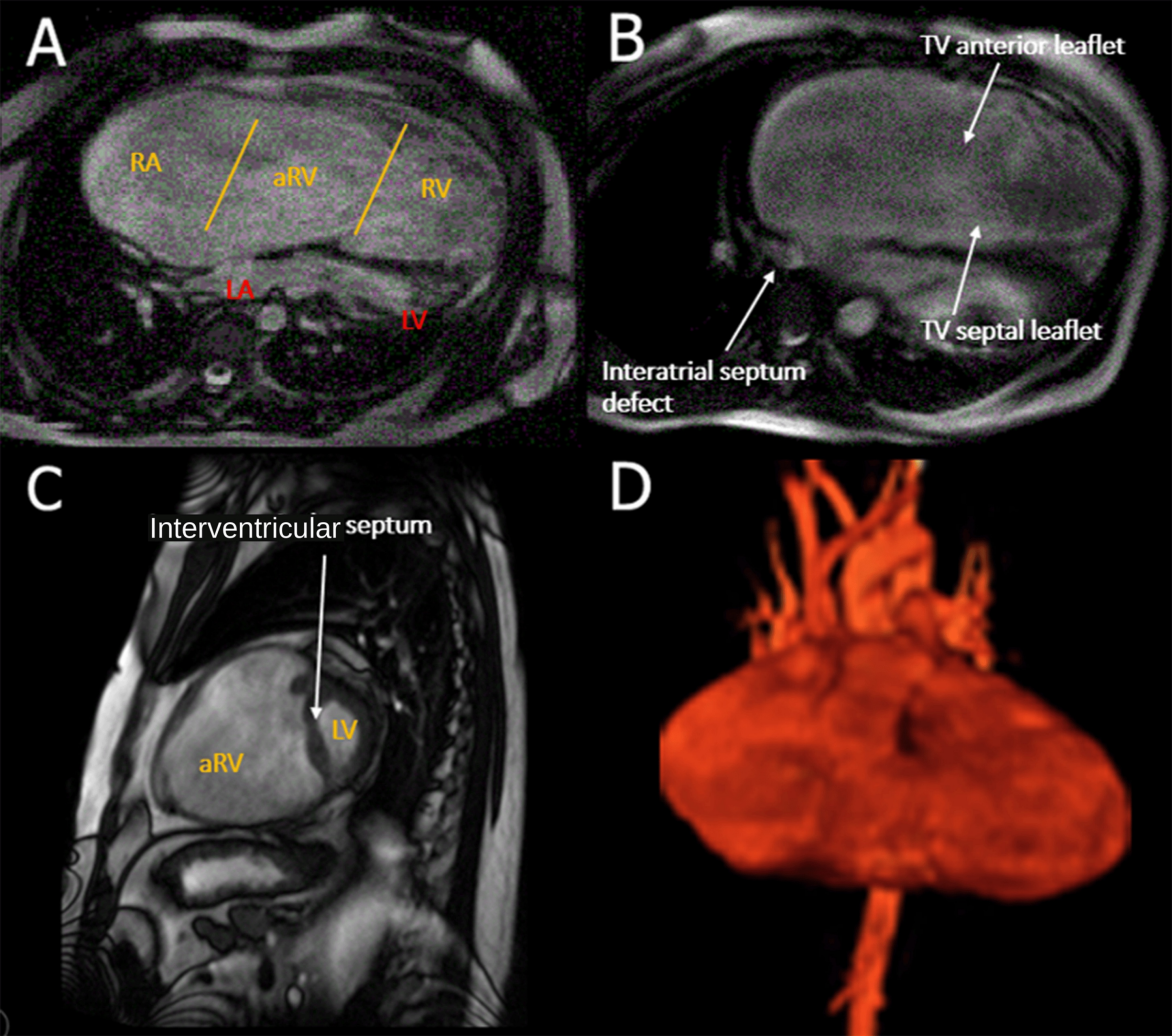
Cardiac magnetic resonance and three-dimensional reconstruction. (A) Four-chamber view showing tricuspid displacement separating the atrialized from the functional right ventricle. (B) Displacement of the anterior and septal leaflets with an interatrial septal defect. (C) Short-axis view showing septal bowing and the atrialized right ventricle (aRV). (D) Three-dimensional computed tomography reconstruction depicting atrial dilation. RA: right atrium; LA: left atrium; LV: left ventricle; TV: tricuspid valve

CMR complements echocardiography and has become the reference method for volumetric and functional assessment. It quantifies RV and right atrial volumes, differentiates the atrialized from the functional RV, and measures functional RV ejection fraction and regurgitant volume; velocity-encoded and cine sequences resolve retrograde flow and ventricular performance, and tissue mapping characterizes myocardial fibrosis, which carries prognostic weight in adults [9,38]. Geerdink and colleagues showed that echocardiography and CMR are complementary rather than interchangeable, and that complete pediatric evaluation often requires both [39]. Representative CMR images of leaflet displacement, the atrialized RV, and an interatrial defect are shown in Figures 8A-8C, with corresponding volumetric parameters in Table 1.

Quantitative and advanced techniques are reshaping how both ventricles are interrogated. Because the geometrically abnormal RV defeats simple ejection-fraction estimates, deformation imaging has gained ground: LV global longitudinal strain detects subclinical dysfunction earlier than ejection fraction and predicts adverse outcome, and right-sided strain is increasingly applied to the functional segment [16,21]. Four-dimensional flow CMR resolves the complex, often multidirectional regurgitant jets and the low, sometimes retrograde, pulmonary flow that simple two-dimensional velocity mapping underestimates, and parametric tissue mapping quantifies the diffuse fibrosis that underlies both ventricular dysfunction and the arrhythmic substrate [15,38]. These reproducible biomarkers are most useful when tracked serially, since the trajectory of right and LV function, rather than any single value, drives the decision to intervene [9].

Computed tomography (CT)

CT is used less often but provides high-resolution anatomy when CMR is contraindicated, when device or pacing hardware precludes magnetic resonance, or when extracardiac and coronary anatomy must be defined before intervention. CT-derived ventricular volumetry correlates with echocardiographic severity indices and can substitute for CMR volumetry in selected patients, and three-dimensional reconstruction depicts chamber enlargement and spatial relationships relevant to procedural planning (Figure 8D) [40]. Because it entails ionizing radiation and iodinated contrast, CT is used selectively; however, modern wide-detector and electrocardiographically gated protocols provide functional as well as anatomical information at acceptable doses and are valuable for coronary and extracardiac assessment before surgery or transcatheter intervention [40].

Diagnostic cardiac catheterization

**Figure 9:**
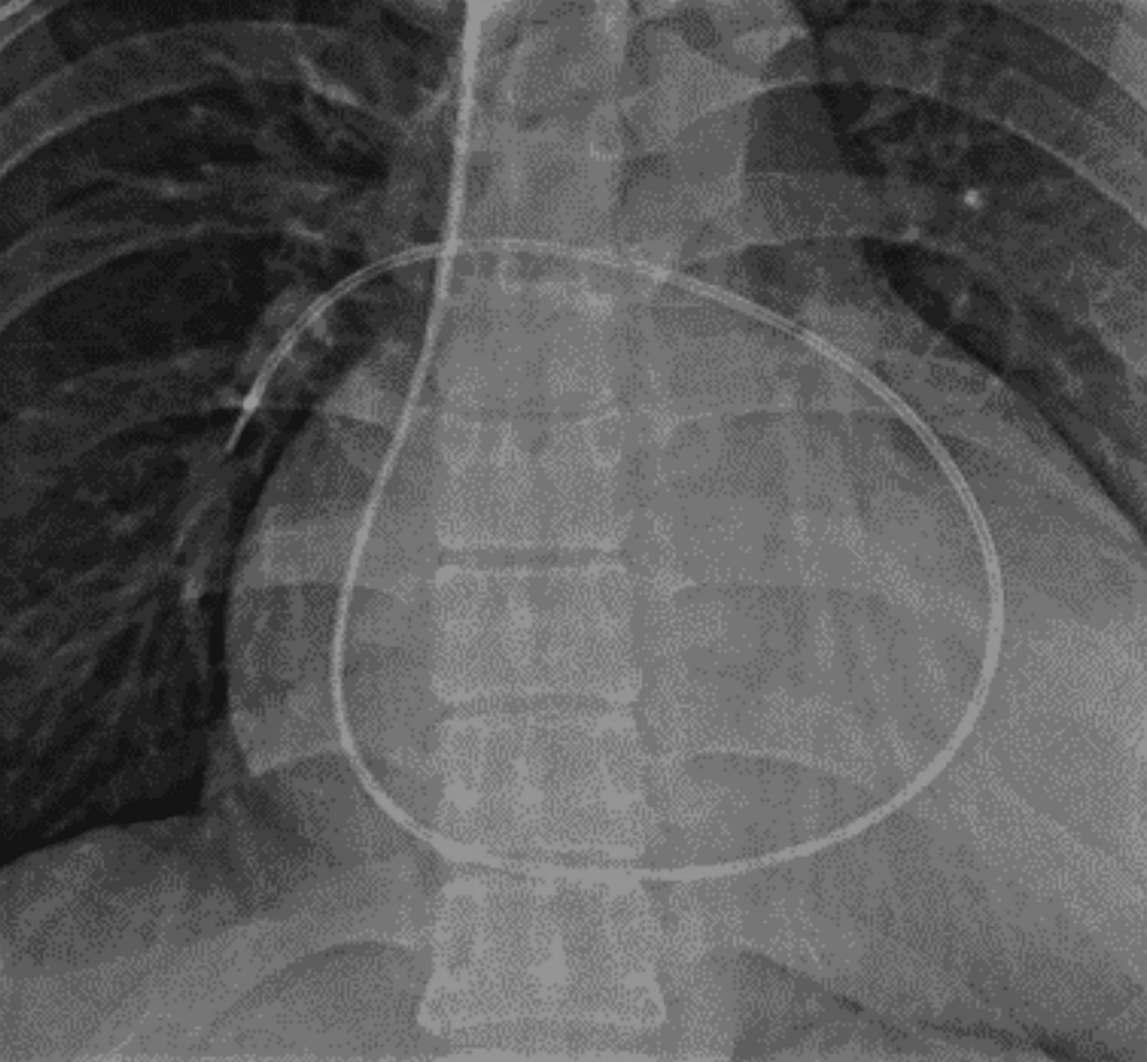
Right heart catheterization. The catheter traverses the dilated right heart chambers into the right pulmonary artery, illustrating the diagnostic (not interventional) use of cardiac catheterization.

Invasive hemodynamic study now has a limited diagnostic role, having been largely supplanted by noninvasive imaging. Diagnostic catheterization remains useful to measure right atrial, right ventricular, and pulmonary arterial pressures, to document oxygen saturations and shunt direction, and to assess pulmonary regurgitation and antegrade pulmonary flow, all of which carry prognostic value; a low preoperative oxygen saturation, in particular, predicts adverse postoperative events; the technique and hemodynamic interpretation of right heart catheterization have been reviewed in detail [41,42]. It is important to distinguish this diagnostic procedure, in which the term cardiac catheterization is conventionally used, from the transcatheter structural and electrophysiological interventions discussed below. A right heart catheter coursing through the dilated chambers of an Ebstein heart is shown in Figure 9.

Each modality carries limitations that temper its role. TTE is operator-dependent and geometrically limited in the crescentic, heavily trabeculated RV, so its volumetric estimates are less reproducible than those for the left ventricle and are best interpreted alongside tomographic data. CMR is the reference standard for biventricular volumes and the tricuspid regurgitant fraction, but it is constrained by cost and limited availability, the need for sedation or general anesthesia in young children, and incompatibility with some pacing or device hardware [43]. CT overcomes this last obstacle and best defines coronary and extracardiac anatomy, yet it entails ionizing radiation and iodinated contrast; in children in particular, its use should be weighed against magnetic resonance and reserved for when magnetic resonance is contraindicated, unavailable, or nondiagnostic. In practice, the modalities are complementary rather than competing, and comprehensive assessment of the pediatric patient often requires both echocardiography and CMR rather than either alone [39].

Management

**Figure 10:**
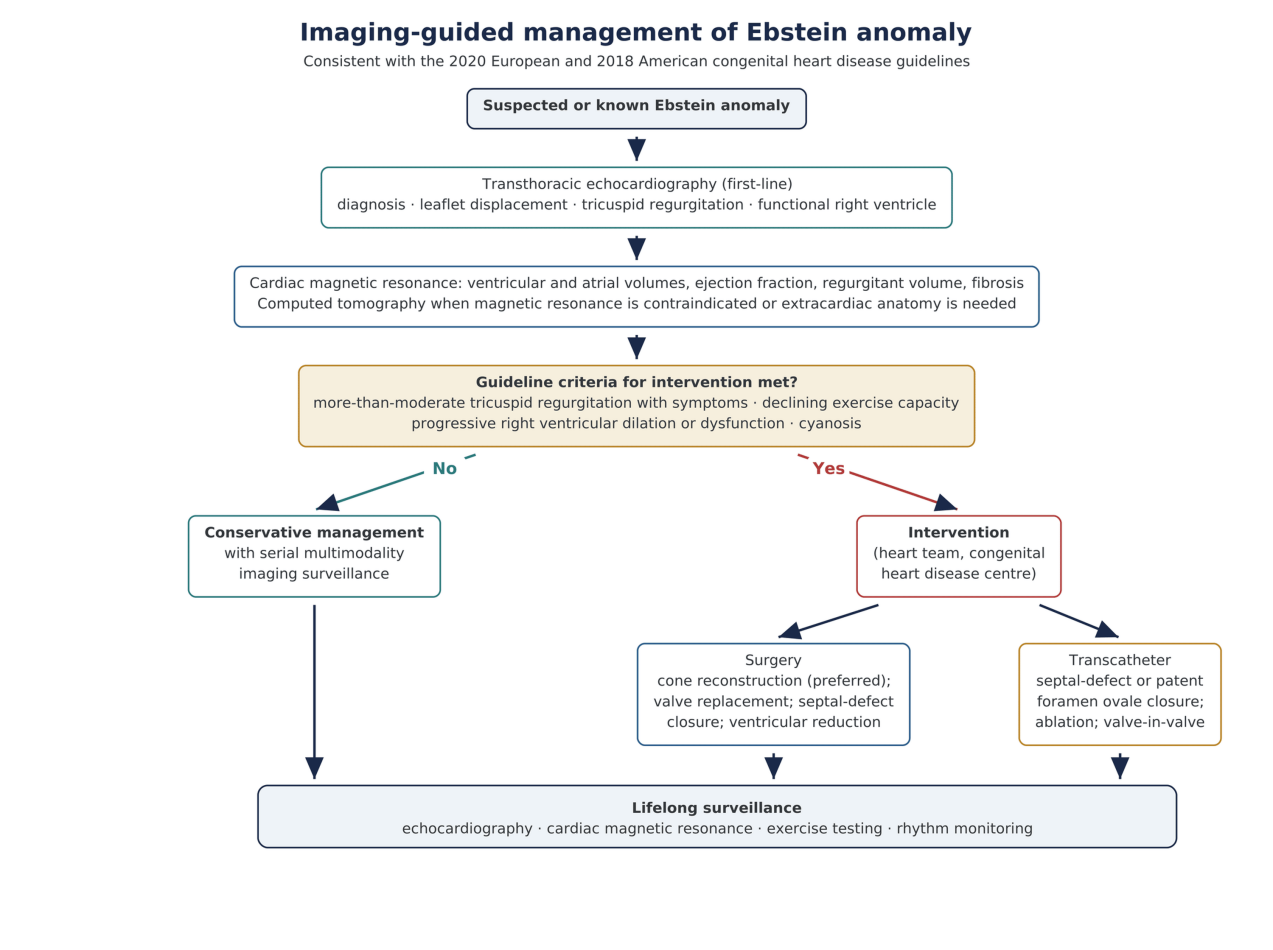
Imaging-guided management pathway. Consistent with the 2020 European Society of Cardiology and 2018 American Heart Association/American College of Cardiology guidelines for adult congenital heart disease, transthoracic echocardiography establishes the diagnosis; cardiac magnetic resonance and computed tomography quantify ventricular volumes, function, and fibrosis; and guideline criteria then direct patients toward conservative surveillance, surgery, or transcatheter intervention, with lifelong imaging follow-up. Image created using the Mind the Graph platform (www.mindthegraph.com; CACTUS Communications, Mumbai, India). The diagrams were completely conceptualized and designed by the authors.

Treatment is matched to symptoms and anatomy and is framed by contemporary practice guidelines. The 2020 European Society of Cardiology guidelines for adult congenital heart disease and the 2018 American Heart Association/American College of Cardiology guideline provide concordant recommendations for Ebstein anomaly: intervention is advised for more than moderate TR with symptoms, deteriorating exercise capacity, progressive RV dilation or dysfunction, or cyanosis, and care should be delivered in centers with congenital heart expertise [44,45]. The recommendations in this review are consistent with both documents; where they differ, it is chiefly in emphasis, with the European guideline providing more explicit thresholds for asymptomatic patients with progressive ventricular change. Indications for surgical and catheter-based treatment are summarized in Table 3, and an imaging-guided treatment pathway is outlined in Figure 10.

**Table 3:** Indications for surgical and catheter-based intervention in Ebstein anomaly, adapted from the 2020 ESC and 2018 AHA/ACC adult congenital heart disease guidelines. Indications are adapted from the 2020 European Society of Cardiology (ESC) and the 2018 American Heart Association/American College of Cardiology (AHA/ACC) guidelines for the management of adult congenital heart disease. Surgical and catheter-based approaches are complementary rather than mutually exclusive; the choice and timing of intervention should be individualized by a multidisciplinary congenital heart disease team after multimodality imaging assessment of valve morphology, right ventricular size and function, and associated lesions. NYHA: New York Heart Association; CPET: cardiopulmonary exercise testing; RV: right ventricle

Indications for surgery	Indications for catheter-based intervention
More than moderate tricuspid regurgitation	Hemodynamically significant accessory-pathway arrhythmia (ablation)
Symptoms beyond NYHA class II or symptomatic arrhythmia	Documented systemic (paradoxical) embolism
Deteriorating exercise capacity on CPET	Predominant cyanosis with oxygen saturation <90% amenable to device closure
Progressive RV dilation, declining RV systolic function, or progressive cardiomegaly	Degenerated bioprosthesis suitable for transcatheter valve-in-valve implantation

A third, more recent document sharpens these thresholds. The 2025 American Association for Thoracic Surgery expert consensus recommends that asymptomatic patients undergo exercise stress testing with measured oxygen consumption to unmask occult exercise intolerance, endorses CMR for biventricular volumes and regurgitant fraction, and considers surgery reasonable in asymptomatic patients with severe regurgitation, moderate right ventricular enlargement, and anatomy favorable for repair [43]. The area of genuine controversy is the timing of intervention in the truly asymptomatic patient, where the balance between pre-emptive repair and watchful waiting remains unresolved and is the subject of ongoing study [43]. On the catheter side, transcatheter tricuspid valve-in-valve implantation is established for the degenerated bioprosthesis, whereas dedicated transcatheter tricuspid replacement and edge-to-edge repair are emerging largely from the broader experience with primary TR and remain investigational in Ebstein anomaly; their eventual role will hinge on anatomical suitability as defined by CT and magnetic resonance imaging [46].

Contemporary recommendations are additionally summarized by age group in Table 4.

**Table 4:** Age-stratified summary of presentation, imaging priorities, and management in Ebstein anomaly. TR: tricuspid regurgitation; GOS: Great Ormond Street; LV: left ventricle; NSAIDs: nonsteroidal anti-inflammatory drugs; TTE: transthoracic echocardiography; TV: tricuspid valve; CMR: cardiac magnetic resonance; CPET: cardiopulmonary exercise testing; CT: computed tomography; ASD: atrial septal defect; PFO: patent foramen ovale; RV: right ventricle

Age group	Typical presentation	Imaging priorities	First-line management	Key considerations
Fetus/neonate	Severe TR, cyanosis, circular shunt, functional pulmonary atresia, hydrops; high perinatal mortality.	Fetal echocardiography: GOS ratio, cardiothoracic ratio, ductal flow direction, functional pulmonary atresia.	Ductal support (prostaglandin) for duct-dependent circulation and supportive care; transplacental NSAIDs in selected circular-shunt fetuses (limited evidence); neonatal repair or single-ventricle palliation for refractory disease.	Prognosis driven by cardiomegaly, hydrops, LV dysfunction, and pulmonary atresia; manage in experienced fetal-cardiac centers.
Infant/child	Variable cyanosis, murmur, exercise limitation, or arrhythmia; some are detected incidentally.	TTE with z-scored TV annulus; increasingly CMR (may need sedation) for biventricular volumes.	Cone reconstruction when indicated - durable, with TV growth and biventricular reverse remodeling; accessory-pathway ablation for symptomatic pre-excitation.	Balance timing against somatic growth and repairability; sedation/anesthesia for CMR; ablation recurrence not negligible.
Adolescent/adult	Asymptomatic for years, or arrhythmia, declining exercise tolerance, or right heart failure.	TTE plus CMR (biventricular volumes, TR fraction, fibrosis); CPET to unmask occult exercise intolerance; CT when CMR contraindicated.	Cone repair preferred (replacement if unrepairable); device closure of ASD/PFO; ablation of documented pathways; valve-in-valve for a degenerated bioprosthesis.	LV status and measured exercise capacity, not resting RV size alone, drive prognosis; lifelong imaging and rhythm surveillance.

Fetal and neonatal therapy

In the fetus with a circular shunt, transplacental non-steroidal anti-inflammatory drugs can constrict the ductus arteriosus and interrupt the retrograde circuit; a systematic review and meta-analysis suggest improved perinatal outcomes compared with expectant management in selected cases, although the evidence remains limited [3]. The critically ill neonate may require stabilization of pulmonary blood flow and, in the most severe anatomy, a single-ventricle pathway when biventricular repair is not feasible [34,47].

This recommendation rests on limited and heterogeneous evidence. The supporting data come from a systematic review and meta-analysis of small observational series comparing transplacental non-steroidal anti-inflammatory drugs with expectant management in fetuses with a circular shunt; the signal toward benefit is encouraging but is drawn from few patients, with attendant risk of bias, and it must be balanced against the recognized fetal hazards of these agents, including constriction of the arterial duct. Transplacental non-steroidal therapy should therefore be regarded as a selective, expert-directed option rather than established treatment, and undertaken only in experienced fetal-cardiology centers.

Surgical interventions

Surgery is indicated for more than moderate TR, symptoms beyond New York Heart Association class II, declining exercise capacity on cardiopulmonary exercise testing, or progressive RV dilation or dysfunction (Table 3) [44]. The objective is a competent tricuspid valve with preservation of native tissue whenever possible. Cone reconstruction, which mobilizes the leaflets and reconstructs a circumferential valve, has become the preferred repair and yields durable improvement in biventricular function and exercise capacity [48-50]. A large single-center experience confirms that results improve with increasing surgical volume, while valve replacement is reserved for valves unsuitable for repair [51]. Closure of an atrial septal defect or patent foramen ovale is performed when paradoxical embolism or cyanosis predominates, often concurrently with valve repair, and right-sided reduction procedures may improve geometry in extreme dilation [52,53].

The durability of cone reconstruction is now supported by comparative and multicenter data. In a single-center comparison of 85 patients, freedom from tricuspid reintervention after cone repair was 97%, 91%, and 91% at two, four, and six years, versus 84%, 74%, and 68% after valve replacement; replacement also carried a higher risk of tricuspid reoperation (37% versus 9%) and late stenosis (21% versus 0%) and worse right ventricular function at last follow-up [54]. A binational Australian and New Zealand series of 125 older children and adults reported 91.5% 10-year survival, a 17% reoperation rate, and no significant survival difference between repair and replacement in a cohort weighted toward complex anatomy [55]. Importantly, residual regurgitation is not abolished: more than mild-to-moderate TR at discharge is more frequent after repair than after replacement, although this early difference does not translate into higher reoperation or mortality by mid-term follow-up [54]. Taken together, these data justify cone reconstruction as the preferred repair while tempering expectations about valve competence, and they argue for continued surveillance rather than an assumption of cure.

Management of the symptomatic neonate remains among the most difficult problems in congenital heart surgery and is individualized. Strategies range from biventricular repair, including neonatal cone reconstruction in experienced centers, to staged single-ventricle palliation when the functional RV is inadequate; the choice depends on the degree of leaflet displacement, the size of the functional RV, and the presence of pulmonary atresia [25,34]. Preoperative oxygen saturation is a useful predictor of the postoperative course [41]. In older children and adults, the timing of elective repair balances the durability of the valve against the risk of irreversible ventricular dysfunction, and contemporary practice favors operating before exercise capacity or right ventricular function declines substantially [44,47]. Surgical practice continues to evolve toward earlier, valve-preserving repair [56].

Transcatheter and electrophysiological interventions

**Figure 11:**
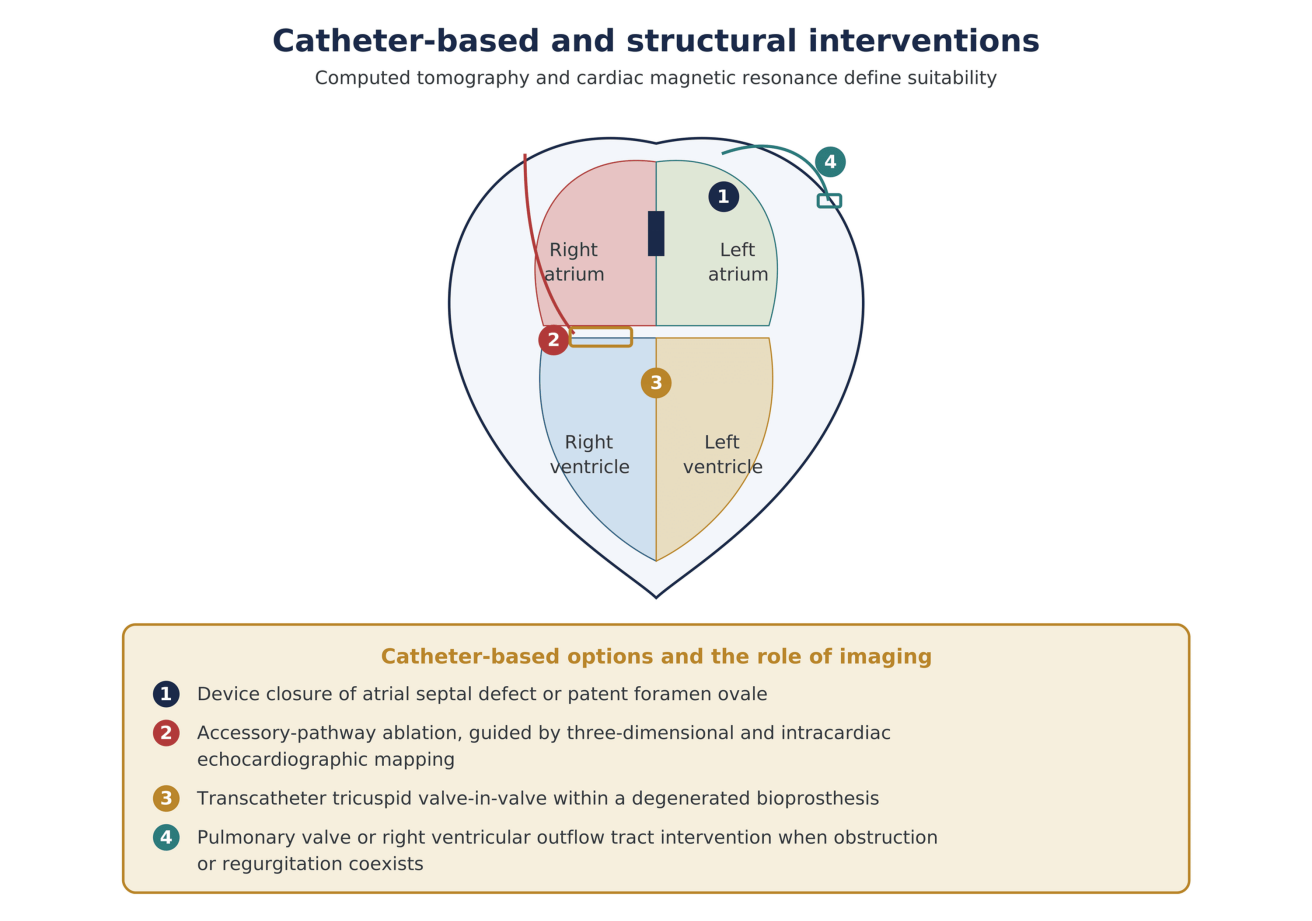
Catheter-based and structural interventions. Device closure of an atrial septal defect or patent foramen ovale, accessory-pathway ablation, transcatheter tricuspid valve-in-valve implantation within a degenerated bioprosthesis, and pulmonary valve or right ventricular outflow tract intervention are shown. Computed tomography and cardiac magnetic resonance define annular dimensions and the regurgitant orifice to judge anatomical suitability. Image created using the Mind the Graph platform (www.mindthegraph.com; CACTUS Communications, Mumbai, India). The diagrams were completely conceptualized and designed by the authors.

Catheter-based therapy is increasingly important for selected patients and for those at high surgical risk, and the role of imaging in selecting and guiding these procedures is expanding (Figure 11) [57].

Radiofrequency ablation, often combined with high-density three-dimensional and intracardiac echocardiographic mapping, is the treatment of choice for accessory-pathway-mediated arrhythmia; the distorted anatomy and frequent multiple pathways make these among the more challenging ablations, and preprocedural imaging of the atrialized region aids mapping [22,23].

The therapeutic yield of ablation warrants explicit appraisal. In Ebstein anomaly, the arrhythmic substrate is often complex - multiple or broad accessory pathways, Mahaim fibers, and a distorted tricuspid annulus - so that a high acute success rate is accompanied by a substantial risk of recurrence. A recent pediatric and young-adult cohort reported accessory-pathway-mediated re-entrant tachycardia as the commonest arrhythmia, an acute ablation success of about 88%, and a recurrence rate of roughly 25%, with arrhythmia and early surgery both bearing on mortality [58]. Contemporary high-density and contact-force mapping aim to improve durability in this difficult substrate. Because arrhythmia contributes materially to late morbidity, rhythm control is best integrated with imaging-based surgical planning - documented accessory pathways addressed before or during cone repair - rather than pursued in isolation.

Percutaneous device closure of an interatrial communication is appropriate for paradoxical embolism, significant cyanosis, or hemodynamic compromise, provided RV function can tolerate loss of the shunt [44]. For the failing bioprosthesis, transcatheter tricuspid valve-in-valve implantation is an established, less invasive alternative to redo surgery; in a multicenter series confined to Ebstein anomaly, valve-in-valve implantation produced good short- and mid-term results and is now a preferred option for degenerated surgical valves [59]. Dedicated transcatheter tricuspid valve repair and replacement systems, developed largely for functional regurgitation in acquired disease, where large real-world registries now confirm high implant success, near-complete regurgitation reduction, and functional improvement [60], are being explored in congenital substrates, but experience in native Ebstein valves remains preliminary and is constrained by the displaced annulus and the large, irregular regurgitant orifice. Interventions on the pulmonary valve and right ventricular outflow tract, including transcatheter pulmonary valve implantation, may be required when outflow obstruction or pulmonary regurgitation coexists. For all of these structural procedures, cross-sectional imaging with CT and CMR is essential to assess annular dimensions, the regurgitant orifice, and the relationship to neighboring structures, and so to judge anatomical suitability [44,57].

Postoperative and long-term surveillance

Lifelong follow-up rests on serial imaging, functional testing, and rhythm monitoring. Echocardiography and CMR assess the integrity of valve repair or replacement, RV function, and atrial size, with attention to residual or recurrent TR, progressive RV dysfunction, and recurrent interatrial shunting; the same modalities that establish the diagnosis are used to detect these complications, as illustrated for TR and ventricular function in Figures 7-8 [39]. Cardiopulmonary exercise testing tracks functional capacity, and ambulatory or implanted monitors detect the late arrhythmias that remain common after repair [21]. Patients with prosthetic valves require attention to anticoagulation and prosthetic valve function, and recurrent regurgitation or worsening RV function may prompt surgical or transcatheter reintervention, including minimally invasive thoracoscopic approaches for recurrent regurgitation after prior repair [51,61]. Follow-up intervals are tailored to severity but are typically annual in the stable adult and more frequent after intervention; surveillance also screens for prosthetic dysfunction, residual shunts, and endocarditis, and CMR is well suited to detecting subclinical right ventricular decline that echocardiography can underestimate [39,50].

Prognosis and outcomes

Outcomes have improved markedly with contemporary surgery and lifelong specialist care, yet prognosis varies as widely as the anatomy. Survival and functional status are governed by the severity of TR, the size and function of the functional RV, the degree of LV impairment, and the arrhythmic burden; LV global longitudinal strain and myocardial fibrosis have emerged as sensitive markers of adverse outcome [15,21,38]. Patients with a well-preserved RV and a durable repair generally enjoy a good quality of life and near-normal life expectancy, whereas advanced right ventricular dysfunction, recurrent regurgitation, or refractory arrhythmia predict limitation and repeated intervention [27,51]. Because deterioration can be insidious, structured follow-up in a congenital heart disease center, anchored by serial imaging, is central to preserving these gains [28].

Reverse remodeling after repair is measurable but incomplete, and it does not guarantee functional recovery. After cone repair, indexed right ventricular end-diastolic volume falls substantially - for example, from 166 to 114 mL/m^2^ in one series - and antegrade stroke volume rises, yet objective exercise capacity on cardiopulmonary testing frequently does not improve, with gains largely confined to the patients who were most symptomatic before surgery [62]. This dissociation between anatomical repair and measured performance mirrors the pathophysiology described earlier: LV underfilling, ventricular interdependence, and myocardial fibrosis constrain a functional reserve that valve competence alone cannot restore. It also reframes surveillance in adults, in whom preserved resting function may coexist with limited exercise tolerance - an argument for incorporating cardiopulmonary exercise testing, rather than resting indices alone, into long-term follow-up [43].

Future directions

The genetic basis of Ebstein anomaly is being clarified, with variants in sarcomeric and cardiac transcription factor genes, including MYH7 and NKX2-5, implicated in a subset of patients, and epigenetic contributions increasingly recognized [63]. Single-cell transcriptomics and genome editing may eventually connect these molecular findings to phenotype. In imaging, the priorities are reproducible biomarkers of RV and LV dysfunction, such as strain and tissue characterization, that can identify the optimal moment for intervention; in therapy, the maturation of transcatheter tricuspid and pulmonary techniques may extend less invasive options to anatomies that today require open or redo surgery [38,57].

## Conclusions

Ebstein anomaly is an anatomically heterogeneous lesion whose management depends on accurate imaging. Echocardiography establishes the diagnosis; CMR quantifies ventricular volumes, function, and fibrosis; and CT assesses anatomy when magnetic resonance is unsuitable; together, they define severity and guide the timing and type of intervention.

Cone reconstruction has improved surgical outcomes, while catheter-based ablation, device closure, and tricuspid valve-in-valve implantation address specific problems in selected patients, in accordance with current European and North American guidelines. Continued progress will come from improved imaging biomarkers, refinement of transcatheter techniques, and care delivered by multidisciplinary teams at centers experienced in congenital heart disease.
